# Epistemology of Natural Strategies for Cardiac Tissue Repair

**DOI:** 10.3389/fcvm.2017.00061

**Published:** 2017-10-02

**Authors:** Michele M. Ciulla

**Affiliations:** ^1^Laboratory of Clinical Informatics and Cardiovascular Imaging, Department of Clinical Sciences and Community Health, University of Milan, Milan, Italy; ^2^Cardiovascular Diseases Unit, Fondazione IRCCS Cà Granda Ospedale Maggiore Policlinico, Milan, Italy

**Keywords:** arrow of time, cardiovascular disease, cellular resources, entropy, environment, information, life, natural strategy, structuring of time

… *there is no science without philosophy*.

## Summary

Is there maintenance in complex biological systems or life is a continuous space-time drift? The emphasis that in the past years has been placed on tissue repair is, at least, misplaced since this property coincides with the same strategy that handles development and maintenance, a strategy that is broadly adaptive. It is not a mere philosophical question, since it deals with limited cellular resources; indeed, the evolution towards multicellularity is a form of adaptation to the environment that involves a high cost in terms of biological resources to handle differentiation, specialization, establishment of functional hierarchies, and development of a complex organisms. It is, therefore, evident that the availability of resources for other forms of adaptation in adult life, such as the re-generation process for maintenance or repair, is limited.

In this opinion, I will discuss the epistemology of natural strategies for cardiac tissue repair, since, in the twentieth century, the increase in life expectancy in some geographic areas has given rise to diseases generally less frequent in the first three or four decades of life; thus, the morbidity and mortality related to cardiovascular disease has shown all the negative aspects of arterial and cardiac ageing bringing to the foreground the problem of cardiac damage and of its repair.

## How to Make Order in the Environment?

Life, in the biological meaning, is the result of continuous interaction with the external environment from which acquires energy and produces order by reducing entropy or disorder (1); since the environment is subject to changes, both cyclical and stochastic, it is logical to assume that biological systems, even the most basic like unicellular ones, can expand *over time* only with an appropriate *natural strategy* to buffer these changes by maintaining performance and a certain stability (2). The tendency of the universe toward disorder—or increase in entropy—must be viewed as a unidirectional process that coincides with what has been defined the *arrow of time*, thus, life is a phenomenon, actually, not separable from the time that passes since is a part of the same *space-time* continuum.

Indeed the changes related to the arrow of time, both internal and external, can be harmful to the living organisms, thus the natural strategy adopted is a responsive *information system* capable to report an injury and, possibly, to initiate and coordinate a reparative response by using a *source code* for the conduct made of bio-molecules. The entropy has been defined as a measure of our *lack of information*, so we can say that the information, encoded from the interaction with the environment as nucleic acids, reduces the level of entropy or, according to Schrödinger (3), produces negative entropy and this is the way how living organisms make order in the universe.

**Figure 1:**
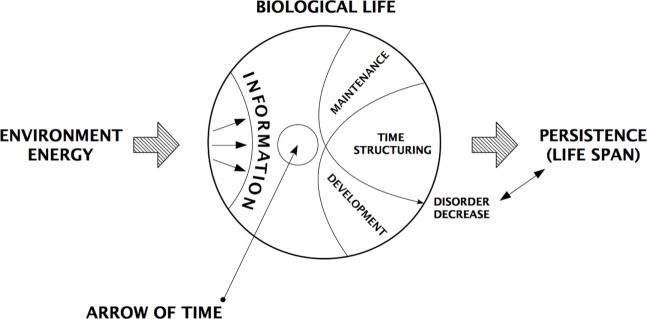
Schematic representation of time structuring that characterizes biological life. The information encoded from the interaction with the environment reduces the level of entropy and make order in the universe.

Regardless of the level of complexity, this information system ensures reproduction, drives development, and manages the ordinary *maintenance of living condition*s for survival, thus the possibility of damages and disease, caused by harmful interactions with the environment, are somehow expected but handled by a unique system that relies on the same biological strategy that is, broadly, adaptive (Figure 1).

## How to Incorporate the Arrow of Time in Biology?

The appearance in an inanimate world of living organisms does not have an explanation that follows the laws of classical physics, thus, it must be admitted a process that involves a certain *degree of persistence* in a far from equilibrium thermodynamic context (4).

Persistence is related to the *structuring of time*, an essential activity that consists in adapting reproduction, development and evolution to time flowing and its rhythms by specific *chronobiological functions*; indeed, biological timing is a measure of the cyclical and stochastic changes rather than an absolute physical quantity (5).

Every living biological unit, regardless of how it is complex, by having embedded the arrow of time, are able to *sense and process internal and external changes* in order to develop a natural strategy for survival and reproduction (6). Thus *processing* is an essential part of what we call biological life that allows, temporarily, to structure time by providing an adaptive response. The *source code* is within the genome that ensures the consistency of information with the *operational scope;* dynamic and flexible responses to changes are ensured through *epigenetic mechanisms* that regulate gene expression of each cell to fix its identity during development and adaptation processes (7).

In such broad sense, the adaptation is an *always on process* featuring life in the biological meaning as a kind of reorganization of environmental *thermodynamic free energy* into organic compounds that are the basis for the *accumulation of information*, from signaling to behavior.

Since it is impossible to reverse the course of events—or entropy—every form of life, has a non-zero probability of dying that increases in a process described as *biological aging*.

In Table 1, the features of biological information processing are summarized.

**Table 1:** Features of biological information processing.

Essential biological function
Uses complex multiple hierarchical encoded networks, from signaling to behavior
Dynamically evolving, interdependent, highly redundant, noisy and resonant
Typically reliable when considering the results
Several key nodes in the network where perturbations may have profound effects

## The Constrain of Having Limited Cellular Resources in Multicellular Organisms

Within the same *adaptive strategy*, the approach to multicellularity introduces a new perspective: the management of—limited*—cellular resources*. Indeed multicellularity involves a high cost, process such as generation, differentiation, and specialization, establishment of functional hierarchies and ontogeny of a fully developed complex organisms implies a remarkable expense of cellular resources, thereby, reducing their availability for other forms of adaptation in adult life, such as regeneration process (8). In organisms with sexual reproduction, which produces genetic diversity, everything starts from (only) two embryonic cells that entrust all their versatility to stem cells. The number of these cells and their *replication cycles* are limited, their potency is maximal during the pre-natal life in the *embryonic stem cells*, and progressively decreases in post-natal life by remaining confined within the *adult stem cells*. Indeed to allow the harmonious development and maintenance of complex multicellular organisms, such as a higher vertebrate, these processes are subject to very tight time constraints: generative and regenerative processes are possible at specific *time-window* through the activation of specific *gene sequences*.

Since the cellular resources available for regeneration are a supply that draws on the same primary or stem cells, we have to admit significant numerical differences between a tissue and another due to the strict *functional hierarchy* that governs the distribution of available stem cells. The very small number of adult stem cells are, thus, unevenly distributed between different tissues, with large numerical differences, as a consequence, even the *regenerative potential* is variable (8).

Many adult tissues contain populations of adult stem cells with *renewal capacity after disease* or during *aging process*, these include brain, bone marrow, peripheral blood, blood vessels, skeletal muscle, skin, teeth, heart, gut, liver, ovarian epithelium, and testis. Thus, *the primary role of adult stem cells in a living organism is to maintain and repair the tissue in which they are found*. Among these tissues, the highly specialized ones express none or very limited regeneration capacity and the reason for this natural strategy is not yet known, even if we can hypothesize that the extreme structural and functional specialization reached is an inherent limit to regeneration.

## Cardiac Tissue in Case of Damage: Regeneration or Repair?

The stem cells supply for the cardiac tissue in mammals is limited and, in any case, even assuming a certain turnover of no more than 1–2% per year for routine maintenance (9), we have to consider that this turnover can reduce the regenerative capabilities of the heart after birth and during aging in case of damage. This gap, which comes from the difficulty of maintaining a certain balance between the demands for ordinary and extraordinary maintenance of cardiac tissue, may not have been a problem for years because, in the past, death occurred before the development of *cardiovascular diseases*, a process that has been proven to take time.

In the twentieth century, the increase in life expectancy in some geographic areas has given rise to diseases generally less frequent in the first three or four decades of life; thus, the morbidity and mortality related to cardiovascular disease has shown all the negative aspects of arterial and cardiac aging (10) bringing to the foreground the problem of cardiac damage and of its repair.

Following a myocardial damage, the loss of myocytes is a matter of fact for the human heart and this loss plays an essential role in the progressive development of *heart failure*; thus, the *natural strategies aimed to the recovery of damaged tissues* are essential tools for the survival of the organism and consists in a *remodeling phase* that is mediated by *epigenetic regulators*. In contrast to the consistency of the genome, the epigenome is characterized by a dynamic and flexible response to different stimuli. The *extraordinary cardiac maintenance* through repair involve a variety of pleiotropic inflammatory mediators whose effects consist, mainly, in initiating and leading the healing process. Among these factors, cytokines are a diverse group of small proteins that play an essential role in *cell signaling* by exhibiting a complex pattern of activation (11). Thus, the cytokines can be considered an essential signaling network devoted to the reporting and management of cellular damage, by modeling the flow of information, they produce order and maintain the cell fate decisions (12).

As stated previously, the reduced availability of regenerative resources in adult life forces to use a *non-original repair tissue* in a process known as *scarring*. It is not known whether if the scarring process itself represents limitations to the regeneration from the surrounding progenitors of cardiac myocytes. Previous studies has shown that the extracellular matrix plays a fundamental role in the maintenance of the complex 3D architecture of the heart acting as a mechanic receptor able to switch-on specific cellular lineages and this, especially, during development (13). This property has been favorably replicated in studies of tissue engineering *in vitro* by using soft and hard layers, with neonatal murine cardiomyocytes, showing that softer layers are able to host mature cardiomyocytes, with fully assembled sarcomeres, while mostly immature figures and upregulation of several genes involved in inflammatory processes were found with the stiffest layers (14). Possibly the latter context is the same that is found *in vivo* in process known as *pathological remodeling* of the heart, including hypertrophy and myocardial damage, where the stiffest collagen is the dominant phenotype, as in the scarring processes, and is followed by an increase in collagen cross-links (15).

## Epilog

In conclusion, the epistemology of natural strategies for tissue repair seems to be deeply tied to the continuum space-time since biological life, although it is extremely improbable, is still highly reproducible, showing a degree of persistence in a far from equilibrium thermodynamic context. The counterpart of having embedded the arrow of time into cellular clocks is quite evident and consists in biological aging, a phenomenon that has to do more with the progressive consumption of limited resources to cope the environment, rather than maintenance and this, especially, in cardiac tissue. As it is actually not possible to reverse the course of time, to describe this process, it seems appropriate to use a neologism which results from the merging of two terms, the first, *spacetime*, refers to the mathematical model that in physics represents the continuum between the three dimensions of space and time, and the second, *drift*, refers to the term used in genetics to describe the variation in gene frequencies following natural or accidental environmental events.

## Author Contributions

The author confirms being the sole contributor of this work and approved it for publication.

## Conflict of Interest Statement

The author declares that the research was conducted in the absence of any commercial or financial relationships that could be construed as a potential conflict of interest.
